# Approach to Identifying Causative Pathogens of Community-Acquired Pneumonia in Children Using Culture, Molecular, and Serology Tests

**DOI:** 10.3389/fped.2021.629318

**Published:** 2021-05-28

**Authors:** Yan Mardian, Adhella Menur Naysilla, Dewi Lokida, Helmia Farida, Abu Tholib Aman, Muhammad Karyana, Nurhayati Lukman, Herman Kosasih, Ahnika Kline, Chuen-Yen Lau

**Affiliations:** ^1^Indonesia Research Partnership on Infectious Disease, Jakarta, Indonesia; ^2^Tangerang District Hospital, Tangerang, Indonesia; ^3^Dr. Kariadi Hospital/Diponegoro University, Semarang, Indonesia; ^4^Dr. Sardjito Hospital/Universitas Gadjah Mada, Yogyakarta, Indonesia; ^5^National Institute of Health Research and Development, Ministry of Health, Republic of Indonesia, Jakarta, Indonesia; ^6^National Institute of Allergy and Infectious Diseases, National Institutes of Health, Bethesda, MD, United States; ^7^National Cancer Institute, National Institutes of Health, Bethesda, MD, United States

**Keywords:** rules, pathogen detection, specimens, bacterial, viral, children pneumonia

## Abstract

Determining the causative pathogen(s) of community-acquired pneumonia (CAP) in children remains a challenge despite advances in diagnostic methods. Currently available guidelines generally recommend empiric antimicrobial therapy when the specific etiology is unknown. However, shifts in epidemiology, emergence of new pathogens, and increasing antimicrobial resistance underscore the importance of identifying causative pathogen(s). Although viral CAP among children is increasingly recognized, distinguishing viral from bacterial etiologies remains difficult. Obtaining high quality samples from infected lung tissue is typically the limiting factor. Additionally, interpretation of results from routinely collected specimens (blood, sputum, and nasopharyngeal swabs) is complicated by bacterial colonization and prolonged shedding of incidental respiratory viruses. Using current literature on assessment of CAP causes in children, we developed an approach for identifying the most likely causative pathogen(s) using blood and sputum culture, polymerase chain reaction (PCR), and paired serology. Our proposed rules do not rely on carriage prevalence data from controls. We herein share our perspective in order to help clinicians and researchers classify and manage childhood pneumonia.

## Introduction

Pneumonia is the leading infectious cause of death amongst children worldwide. It accounts for more than 138 million new cases and almost one million deaths annually, mostly amongst children under 5 years old (1, 2). Research on pneumonia etiologies conducted from the 1970s through the early 1990s showed 2 bacteria—*Streptococcus pneumoniae* and *Haemophilus influenzae* type b (Hib)— cause the majority of fatal pneumonia cases in children, primarily in settings that lack access to basic healthcare such as antibiotics and oxygen therapy (3, 4). The World Health Organization (WHO) used these data to develop a clinical case definition for pneumonia, which deliberately increased sensitivity to ensure that all potential pneumonia cases would receive effective antibiotic therapy (5, 6).

In line with WHO guidelines, practice guidelines from the British Thoracic Society (BTS) in 2011 and Royal College of Paediatrics and Child Health (RCPCH) in 2016 recommend amoxicillin as the first choice for oral antibiotic therapy in children with community-acquired pneumonia (CAP) (7–9). The 2011 Pediatric Infectious Diseases Society/Infectious Diseases Society of America (IDSA/PIDS) guideline recommends narrow-spectrum antibiotics (e.g., amoxicillin or amoxicillin-clavulanate) for most children hospitalized with CAP, and macrolides (azithromycin, clarithromycin, or erythromycin) for presumed atypical pathogens (10). Despite evidence that appropriate antibiotics can be lifesaving, rational selection of antibiotics for pneumonia is hampered by low adherence to existing guidelines and scarcity of point-of-care diagnostics (11, 12). Consequently, healthcare providers, particularly those in low-resource settings, are likely to overtreat non-bacterial pneumonia with antibiotics (11).

Overuse of antibiotics can engender resistance to multiple antibiotic classes as well as toxicity. Recent CAP etiology studies, including the large-scale Pneumonia Etiology Research for Child Health (PERCH) and the Global Approach to Biological Research, Infectious diseases and Epidemics in Low-income countries (GABRIEL) studies, have focused on low-income countries (13, 14). They reveal that viral etiologies of CAP in those settings have likely been underestimated due to prior lack of viral diagnostics, shifting pathogen prevalence associated with widespread deployment of Hib and pneumococcal conjugate vaccines (PCV), improved socioeconomic and nutritional status, a sharp decrease in measles incidence, and increased urbanization (3, 15). Thus, empiric antibiotic treatment algorithms for pediatric CAP may need updating (3, 15).

Identification of specific viral and bacterial causes of pneumonia is hindered by non-specificity of clinical and radiographic findings, as well as co-infection and colonization (16). Nasopharyngeal carriage of respiratory bacteria and viruses known to be associated with pneumonia have been reported in apparently healthy children, obscuring their contribution to pneumonia in many cases (17–19). To address this diagnostic challenge, many pneumonia studies include a control group of healthy children (13, 14, 19, 20). Inclusion of control children reduces over-attribution of disease to non-pathogenic organisms by allowing calculation of an adjusted odds ratio (aOR) for each pathogen and estimation of the population-attributable fraction (13, 19). However, obtaining lower respiratory tract specimens from healthy control children, though ideal for study design, may not be feasible in certain situations (21) and is difficult to accomplish in clinical practice.

## Our Approach

In order to interpret data collected for a pediatric pneumonia study in Indonesia, rules for assessing relevance of identified organisms were needed. The study, known as “Partnerships for Enhanced Engagement in Research - Pneumonia in Pediatrics (PEER-PePPeS)” was a multi-site observational cohort study that sought to estimate etiologies of CAP amongst children aged 2–59 months of age in Indonesia, where PCV and Hib vaccines are not mandatory (https://sites.nationalacademies.org/PGA/PEER/PEERscience/PGA_174220) and all CAP cases are treated with empiric antibiotics per national guidelines (22). PEER-PePPeS aimed to identify the causative pathogen(s) and etiologic distribution of pneumonia cases via comprehensive diagnostic testing across three research sites. Diagnostics were selected from assays commonly performed in clinical practice, with addition molecular and paired-serology tests in PEER-PePPeS for a comprehensive approach.

**Figure 1:**
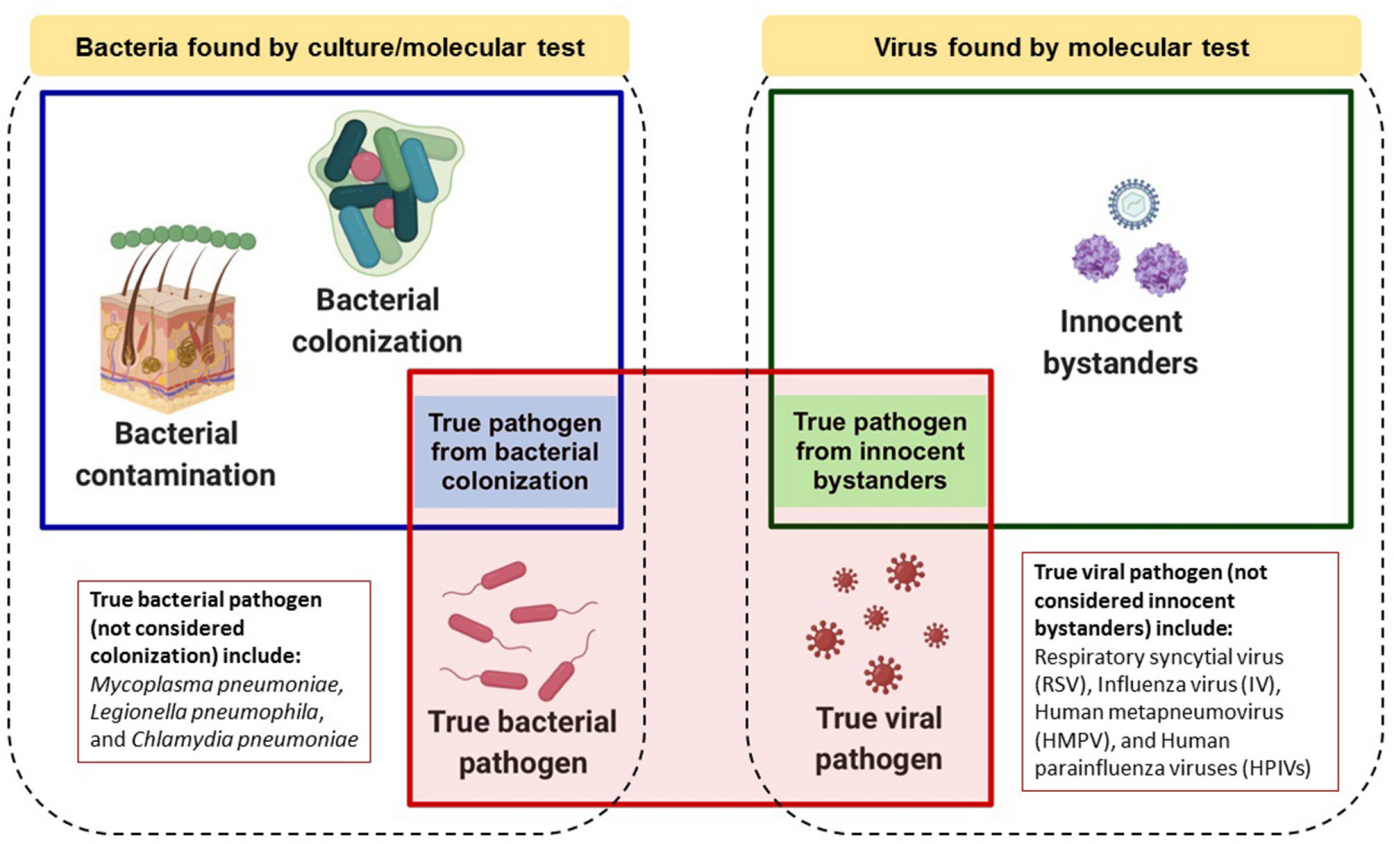
Conceptual model of roles that bacteria or viruses detected by laboratory testing might play in childhood pneumonia. Dotted boxes encompass all bacteria or viruses found by microbiologic and/or molecular testing. Blue or green boxes represent bacteria or viruses, respectively, which could be causative pathogens. The red box represents true causative pathogens identified by the proposed “rules.” Image was created in Biorender.com.

Current literature was considered during formulation of the rules. PubMed search terms “children,” “pneumonia,” “bacterial pathogen,” “colonization” or “carriage,” “viral pathogen,” “innocent bystanders,” “specimen type,” “culture,” “molecular” and “serology” were used. Additionally, large-scale, case-control studies conducted in developing countries, such as PERCH and GABRIEL, and expert opinion in Indonesia were taken into account. A conceptual summary of the proposed rules is depicted in Figure 1 and further explained in this paper.

### Specimen Types and Detection Methods

Determination of pneumonia etiology is ideally based upon isolation of causative pathogens, or characteristic histology in the case of non-infectious pneumonia, at the site of disease (23). However, effective sampling from the lower respiratory tract (LRT) is frequently not clinically feasible. Upper respiratory tract (URT) specimens such as nasopharyngeal (NP)/oropharyngeal (OP) swabs must often serve as a proxy. These URT specimens can be particularly difficult to interpret due to confounding by asymptomatic carriage of non-pathogenic organisms (24).

The nasopharynx is a complex and dynamic environment; any organism that could cause pneumonia may also be a colonizer, including *Streptococcus pneumoniae, Haemophilus influenzae, Staphylococcus aureus*, and *Moraxella catarrhalis* (17, 25, 26). Furthermore, asymptomatic NP carriage may responsible for transmission and often precedes LRT infection (27). *Klebsiella pneumoniae* is an opportunistic gram-negative bacteria that can colonize the NP, although it more commonly colonizes the gut (28, 29). Studies have reported a lower prevalence of NP carriage in children (range 1.4–7%) than in adults (20–32.5%), which may be related to poor hygiene (29–31). However, in terms of its pathogenicity, the presence of *K. pneumonia* in symptomatic children should be considered a pathogen (28, 32).

Previous studies have suggested that children with pneumonia have a higher pathogen density in the URT than children without pneumonia, though there is heterogeneity by study and pathogen (25, 33). Likewise, respiratory viruses found in respiratory specimens by molecular tests during acute disease may be “innocent bystanders” without a causal role (34). The presence of a respiratory virus, such as human Bocavirus (hBoV), Adenovirus (AdV), non-SARS human Coronavirus (hCoV), Enterovirus (EV), and Rhinovirus (RV), in the URT of healthy children has been observed in several studies (13, 19, 20). This could represent carriage, nascent infection, prolonged shedding, or subclinical infection (18, 34, 35). Some respiratory viruses, such as hBoV, can shed for several months after an illness (36).

Secretions from the LRT are also diagnostically helpful as they originate at the site of infection (24). However, children have difficulty expectorating sputum, leading to frequent use of induction techniques to obtain LRT specimens. Sputum induction is performed by administration of hypertonic saline via nebulizer, followed by percussion of the chest wall to mobilize secretions (21). The presence of <10 squamous epithelial cells (SECs) and significant number of polymorphonuclear cells/PMNs (for which criteria vary among studies) per low-power field (100× magnification) have long been regarded as ideal (24, 37). However, the criterion of large numbers of PMNs has been questioned given that pneumonia may not necessarily be associated with production of purulent sputum (38). Recent studies showed <10 SECs is the key quality measure in children since it corresponds with low quantities of NP/OP flora (38, 39). However, interpretation should take into account that contamination may occur even with meticulous technique (24).

Microbiologic analysis of LRT specimens, including induced sputum, by techniques such as culture and gram stain can reveal an etiology and guide targeted management (21, 40). Polymerase chain reaction (PCR) for bacterial DNA by is particularly helpful in culture-negative cases, highlighting the complementary role of molecular methods (41). Results should be interpreted in the context of immune status, as opportunistic pathogens such as *Pneumocystis jirovecii* and cytomegalovirus (CMV) are often pathogenic in children with HIV but not in HIV-uninfected children (42).

Etiologic assessment of pneumonia may also be done using blood samples, which may be easier to collect than LRT specimens and NP swabs. As blood is normally sterile, culture of an organism is generally considered indicative of active infection (24). However, prevalence of blood culture contamination may be as high as 2–3% (43) and bacteremia only occurs in 2.1% of pneumonia cases (44). Thus interpretation must account for the possibility of contamination, which we have incorporated in our pathogen identification rules. Blood specimens may also undergo molecular testing. This is especially helpful for some organisms. For instance, PCR detection of *S. pneumoniae* in blood may be associated with invasive pneumococcal disease (44, 45).

Antigen detection on several specimen types, including urine, pleural fluid and NP/OP swabs, is commonly used for point-of-care (PoC) diagnosis (23). Although antigen detection tests are only available for select organisms, they can play a critical role in treatment decisions. For example, rapid recognition of *S. pneumonia* or *Legionella pneumophila*, can guide antibiotic selection. A limitation of antigen detection assays is their reliance on detectable quantities of antigen; performance is thus suboptimal compared with molecular tests (45).

Serological testing of paired acute and convalescent samples, typically collected at least 7 days apart, can also be used for pathogen identification (23). Serology is most helpful for retrospective confirmation, especially for fastidious bacteria, and may reveal the accuracy of a previous empiric diagnosis (45). It may be particularly useful for viruses with prolonged NP shedding or high prevalence in a control population (24). IgM only tests are less sensitive and specific than IgG and IgM antibody titers from paired specimens as IgM kinetics vary (23) and IgM seroconversion might not occur in the setting of repeat infection (46). A serologic diagnosis generally requires a 2-fold or greater increase in titers between paired serum specimens. Reliance on convalescent specimens means that actionable results are not typically available during acute illness, limiting application in the acute-care setting (47).

### Proposed Rules to Assess Bacterial Pathogens

Our rules to determine the bacterial etiology of pneumonia incorporate commonly collected specimens and routine laboratory assays. Blood culture and whole-blood PCR are considered first-tier due to high specificity, followed by induced sputum culture and molecular testing (PCR) of NP/OP swabs or induced sputum specimens (45). Serological evaluation of paired acute and convalescent specimens is also used for diagnosis, especially of atypical bacterial agents (48) for which PCR showed low sensitivity and poor concordance compared with the paired serology (49). The proposed pathogen determination rules are shown in Table 1.

**Table 1:** Rules for identification of causative pathogens in childhood pneumonia.

**Laboratory methods**	**Specimen(s)**	**Bacterial rules**	**Viral rules**
Blood culture/blood PCR	Whole blood	1.All organisms detected by blood culture, except for contaminants^a^, are considered potential pathogens. 2. Any non-contaminant bacteria found by PCR is a potential pathogen.	Not applicable.
Sputum culture and gram stain	Induced sputum	1.A good quality specimen is required, as defined by <10 squamous epithelium per low-power field (magnification, 100×). 2. An organism isolated in quantities of 2+ or 3+ and with compatible Gram stain morphotype is regarded as the pathogen	Virus culture is not routinely done.
Molecular test (PCR)	Nasopharyngeal/Oropharyngeal Swab (NP/OP) and/or Induced Sputum	1.For bacteria not classified as NP colonizers^b^, any positive PCR indicates a pathogen. 2. For bacteria that can be colonizers^c^, higher density is considered indicative of causality if the copy number exceeds 6.9 log_10_ copies/mL for *S. pneumoniae*, 5.9 log_10_ copies/mL for *H. influenzae*, and 7.5 log_10_ copies/mL for *S. aureus*. Serodiagnosis is also acceptable (see below).	1.A PCR test with Ct value ≤ 40 is considered diagnostic for viruses known to cause pediatric pneumonia^d^. 2. For some innocent bystander viruses^e^, only those with high viral load (Ct value <24) are regarded as true pathogens. Serodiagnosis is also acceptable for diagnosis (see below).
Serologic Test	Paired serum (acute-convalescent)	1.Initial detection of specific antibodies when the preliminary sample is negative (seroconversion). 2. A two to four-fold increase, depending on the test, in antibody titers in the convalescent specimen.	1.Initial detection of specific antibodies when the preliminary sample is negative (seroconversion). 2. A two to four-fold increase, depending on the test, in antibody titers in the convalescent specimen.

a *List of contaminant bacteria: Coagulase-negative staphylococci, Micrococcus spp., Propionibacterium spp., Alpha-hemolytic streptococci (except pneumococcus, Streptococcus anginosus, and Streptococcus mitis), Enterococcus spp., Corynebacterium spp. (diphtheroids), Bacillus spp. (except Bacillus anthracis), Pseudomonas spp. (except Pseudomonas aeruginosa), Stomatococcus, Aeroccocus, Neiserria subflava, Veillonella spp., other environmental non-fermenting Gram-negative rods, and Candida spp*.

b *List of non-colonizer bacteria: Mycoplasma pneumoniae, Legionella pneumophila, and Chlamydia pneumonia*.

c *List of colonizer bacteria: Streptococcus pneumoniae, Haemophilus influenzae, and Staphylococcus aureus*.

d *List of viruses well-known to cause pediatric pneumonia: Respiratory syncytial virus (RSV), Influenza virus (IV), Human metapneumovirus (HMPV), and Human parainfluenza viruses (HPIVs)*.

e *List of innocent bystander viruses: human Bocavirus (hBoV), Adenovirus (AdV), non-SARS human Coronavirus (hCoV), Enterovirus (EV), and Rhinovirus (RV)*.

All bacteria detected by blood culture, except for contaminants, are considered potential pathogens. The following were classified as contaminants based on relatedness to community-acquired pneumonia: Coagulase-negative staphylococci, *Micrococcus* spp., *Propionibacterium* spp., Alpha-hemolytic streptococci (except pneumococcus, *Streptococcus anginosus*, and *Streptococcus mitis*), *Enterococcus* spp., *Corynebacterium* spp. (diphtheroids), *Bacillus* spp. (except *Bacillus anthracis*), *Pseudomonas* spp. (except *Pseudomonas aeruginosa*), *Stomatococcus, Aeroccocus, Neiserria subflava, Veillonella* spp., other environmental non-fermenting Gram-negative rods, and *Candida* spp. (14, 20). Any bacteria found in whole blood by PCR and not listed as a contaminant is classified as a pathogen (20).

Good quality sputum specimens should undergo gram stain and bacterial culture simultaneously to optimize microbiological yield (21, 50). Gram stain results that are consistent with sputum culture can define the microbiological diagnosis of pneumonia (21). The gram stain should be interpreted as follows: Gram-positive lancet-shaped diplococci (GPDC) suggest *S. pneumoniae*; Gram-positive diplococci (GPDC) or cocci in chains suggest *Streptococcus pyogenes*; Gram-positive cocci in clusters (GPC-cluster) suggest *S. aureus*; Gram-negative coccobacilli (GNCB) suggest *H. influenzae, Bordetella pertussis* or *Acinetobacter baumannii*; Gram-negative diploccoci (GNDC) suggest *M. catarrhalis*; large Gram-negative rods (GNR-large) suggest *Klebsiella pneumoniae* or *Escherichia coli*; and small Gram-negative rods (GNR-small) suggest *P. aeruginosa* (40).

For semiquantitative measurement on sputum culture, predominant organisms were identified and quantified according to the furthest quadrant with visible colonies (first quadrant, scanty; second quadrant, 1+; third quadrant, 2+; fourth quadrant, 3+). Organisms isolated in quantities of 2+ or 3+ and with compatible Gram stain morphotype are regarded as pathogens (38). For quantitative culture, colony counts of <10^4^/ml suggest contamination, counts of 10^4^-10^5^/ml are indeterminate, and counts of >10^5^/ml of a major isolate suggest a potential pathogen (51). If the organism grows on culture, conventional biochemical tests (e.g., catalase or coagulase) or automated system evaluations, including rapid fluorescence-based methodology, can be performed to identify the organism (52, 53).

Culture yield depends on sputum quality, pathogen burden, transport time, storage period, nutrients, and incubation conditions that maintain viability (50, 54). Receipt of antibiotics before specimen collection may prevent growth on culture (41) as well as affect gram stain morphology. In this setting, culture-negative bacteria should remain in the differential and be sought by a molecular method targeting DNA (41). For bacteria identified by PCR of respiratory specimens (NP, OP or induced sputum), careful consideration must be given to distinguishing colonizers from true pathogens (14, 17).

For bacteria not considered NP colonizers (e.g., *Mycoplasma pneumoniae*), any positive PCR indicates a definite pathogen (13, 19). For bacteria than can be both a colonizer and pathogen, higher density has been associated with pathogenic status. For example, higher density is associated with causality and considered significant if the copy number exceeds 6.9 log_10_ copies/mL for *S. pneumoniae* (26), 5.9 log_10_ copies/mL for *H. influenzae* (25), and 7.5 log_10_ copies/mL for *S. aureus* (33). Copy number cut-offs were transformed to corresponding Ct values per our assay; the Ct cut-off for *S. pneumonia* was ≤ 24.2; *H. influenzae* ≤ 30.3 and *S. aureus* ≤ 30.2. We nonetheless recognize that Ct value cut-offs may differ depending on the PCR system used. Lower copy numbers are considered indicative of colonization. However, studies have failed to identify an association between density and pathogen-confirmed pneumonia for *M. catarrhalis*, indicating that PCR alone cannot be used to determine its role in childhood pneumonia (25, 33).

Despite limited use in acute management of pneumonia, serological techniques are heavily utilized in clinical research (45). In comprehensive epidemiology studies, serology may identify infections missed by other methods such as culture and PCR (45) and could also distinguish colonization since nasopharyngeal carriage does not cause seroconversion between paired sera (55). A study in children over 2-years old throughout Asia demonstrated that a combination of paired serology, direct antigen and DNA tests increases diagnostic yield of atypical pathogens such as *M. pneumoniae, Chlamydia pneumoniae*, and *Legionella pneumophila* (56).

For interpretation of paired serologic testing, the following are considered indicative of infection: (a) Initial detection of specific antibodies after a prior negative sample (seroconversion), or (b) A two to four-fold increase, depending on the test, in antibody titers in the convalescent specimen (47). Serologic confirmation of pneumonia etiology is especially useful in prolonged hospitalization/ICU admission cases (57), and epidemiologic research or disease surveillance, as opposed to the acute clinical setting (45).

At present, few antigen detection assays are available for diagnosis of bacterial causes of childhood pneumonia. Notable examples include antigen detection assays for *S. pneumoniae and L. pneumophila* (45). The immunochromatographic urine test used for pneumococcal disease detects the polysaccharide cell wall antigen (58). Unfortunately, the test is not reliable in children since it cannot distinguish between carriage and pathogenic pneumococcus (59). Likewise, detection of soluble Legionella antigen in urine is limited to *L. pneumophila* serogroup 1 (60). Given their low sensitivity compared with PCR testing, use of these antigen assays in children is not recommended (45) and not included in our proposed rules.

### Proposed Rules to Assess Viral Pathogens

Detection of viruses by direct immunofluorescence microscopy and isolation in cell culture have long been considered the “gold standard” of respiratory viral pathogen diagnosis (45). However, viral culture is resource-intensive, requires maintenance of cell-lines over long time periods, and poses exposure risk to laboratory personnel (61). Direct fluorescent-antibody (DFA) and immunofluorescent-antibody (IFA) assays of cell smears to detect specific viruses are commercially available and use standardized reagents (e.g., FLUA/B, PIVs 1 to 3, ADV, hMPV, and RSV), but are labor-intensive, require a fluorescence microscope and skilled microscopist, and are susceptible to reader error (62). Therefore, these “gold standard” methods are being replaced by more sensitive, high-throughput, and less labor-intensive molecular tests (45, 48).

A PCR test on NP/OP swabs or induced sputum with Ct (cycle threshold) value ≤ 40 is considered diagnostic for viruses well-known to cause pneumonia, including Respiratory syncytial virus (RSV), Influenza virus (IV), Human metapneumovirus (HMPV), and Human parainfluenza viruses (HPIVs) (13, 19). However, some viruses normally detected in healthy children or that have prolonged/intermittent shedding (innocent bystanders), including RV (63), AdV (64), hBoV (65), non-SARS hCoV (13), and EV (66), merit additional justification. To avoid attributing disease to innocent bystanders, we consider only those with high viral load (Ct value <24) as true pathogens (18, 67).

Viruses confirmed by serodiagnosis are also regarded as causative pathogens. Serology may be particularly useful for identifying respiratory viral infections that trigger secondary bacterial pneumonia as the virus may no longer be detectable in respiratory specimens (45). Addition of serology to PCR evaluation increases positive diagnostic yield (68). In one study, 29 of 88 cases of viral pneumonia were diagnosed by serology alone (69). Serology is a helpful adjunct to the PCR in assessing etiologic contribution, but may not reveal exact timing (68).

Antigen testing for respiratory specimens is attractive as a potential PoC diagnostic test for viral causes of childhood pneumonia. It has been used for detection of seasonal influenza and RSV (45). However, performance is suboptimal; sensitivity of rapid diagnostic tests ranges from 10 to 96% for influenza (70, 71) and 71–95% for commercial RSV (72, 73). Variable sensitivity is not surprising, as low viral loads would predispose to false negatives. Furthermore, low pre-test probability outside respiratory virus season undermines predictive utility (62). Antigen destruction may occur with freezing and on repository swabs, which can also decrease accuracy (74). Based on sensitivity, antigen testing is inferior to PCR for detection of viruses causing pneumonia (62) and is not included in our rules.

## Conclusion

In conclusion, identifying bacterial and viral etiologies of pneumonia in children is necessary yet challenging. NP colonization and prolonged shedding of innocent bystander respiratory viruses may obscure assay interpretation. Our rules employ culture, molecular, and serology testing for determination of the pathogen(s) causing pneumonia in children. These rules will support effective clinical management of and research on childhood pneumonia. Our proposed rules are advantageous in their comprehensive approach, which may increase accuracy of diagnosis. This is very useful for research and well-equipped facilities. However, cost and complexity may present barriers in many laboratories and hospitals, limiting feasibility in low-resource settings. Additionally, reliance on paired serum (acute-convalescent) for assessment could delay diagnosis. Further studies should be performed to assess the utility of these proposed rules for reducing antimicrobial use and resistance rates, as well as correlation with biomarkers used to guide treatment (e.g., procalcitonin, C-reactive protein).

## Data Availability Statement

The original contributions presented in the study are included in the article/supplementary material, further inquiries can be directed to the corresponding author/s.

## Author Contributions

YM, AM, C-YL, HK, and DL prepared the concept and design of the study. YM, AM, HK, and C-YL reviewed and analyzed the literature. YM and AM drafted the manuscript. YM, AM, HK, C-YL, AK, and DL reviewed the draft of the manuscript, provided critical insights, edited and prepared the final version of the manuscript. YM, AM, DL, HF, AA, NL, MK, HK, AK, and C-YL analyzed, reviewed, and edited the manuscript's final version and approved it for publication. All authors contributed to the article and approved the submitted version.

## Conflict of Interest

The authors declare that the research was conducted in the absence of any commercial or financial relationships that could be construed as a potential conflict of interest.
